# Genes and quantitative genetic variation involved with senescence in cells, organs, and the whole plant

**DOI:** 10.3389/fgene.2015.00057

**Published:** 2015-02-23

**Authors:** Benoit Pujol

**Affiliations:** ^1^CNRS, Université Paul Sabatier, ENFA, UMR5174 EDB (Laboratoire Évolution et Diversité Biologique)Toulouse, France; ^2^Université Toulouse 3 Paul Sabatier, CNRS, UMR5174 EDBToulouse, France

**Keywords:** aging, ROS, SAG, SEN, PAG, additive genetic variation, mutation accumulation, antagonistic pleiotropy

## Abstract

Senescence, the deterioration of morphological, physiological, and reproductive functions with age that ends with the death of the organism, was widely studied in plants. Genes were identified that are linked to the deterioration of cells, organs and the whole plant. It is, however, unclear whether those genes are the source of age dependent deterioration or get activated to regulate such deterioration. Furthermore, it is also unclear whether such genes are active as a direct consequence of age or because they are specifically involved in some developmental stages. At the individual level, it is the relationship between quantitative genetic variation, and age that can be used to detect the genetic signature of senescence. Surprisingly, the latter approach was only scarcely applied to plants. This may be the consequence of the demanding requirements for such approaches and/or the fact that most research interest was directed toward plants that avoid senescence. Here, I review those aspects in turn and call for an integrative genetic theory of senescence in plants. Such conceptual development would have implications for the management of plant genetic resources and generate progress on fundamental questions raised by aging research.

Senescence is not a simple concept. Senescence is the decline of organismal functions with age. For physiologists, it can be defined as the degradation of cells or organs and their associated functionality. For demographers, senescence is defined by the positive relationship between the probability to die and age. For evolutionary biologists, senescence is also defined as a decline in function and an increase in the chance of dying with age. Their focus is, however, made on the decline in the strength of natural selection with age at the population level. In the present review, my aim is not to discuss the knowledge accumulated on plant senescence in every discipline. It is not to animate a semantic debate for the sake of providing a unique definition of senescence. Here, my focus is on genetic data and its involvement with plant senescence. I will explore that question at the level of cells, organs, and individuals within populations. I will then discuss how recent methodological developments in quantitative genetics could be used to develop an integrative framework and investigate the links observed between those scales.

## GENES INVOLVED WITH CELL SENESCENCE

Senescence at the cellular level is the deterioration of the cell that is due to the age of the cell and not to the age of the whole organism. In plant cells, as in animal cells, life in aerobic conditions induces the production of reactive oxygen species (ROS) such as hydrogen peroxide (H_2_O_2_), and nitric oxide (NO). They are toxic and despite the action of antioxidant mechanisms, their toxicity leads to the death of cells (Van Breusegem and Dat, 2006). Their production is inevitable because they are the consequence of membrane linked electron transport in plant cells. ROS are generated during the production of energy through respiration and photosynthesis. In *Arabidopsis* cells, the expression level of the gene *LSC54* was found to increase with the age of cells. The activity of *LSC54* was also found to be linked with the level of oxidative stress in the cell. One might think that the activity of *LSC54* is generating oxidative stress but in fact, the activity of *LSC54* is induced by increased levels of ROS (Navabpour et al., 2003). Such genes probably become active in response to ROS signaling in order to regulate oxidative mechanisms of deterioration.

Many genes are involved with cell senescence. They can be found under the name of senescence associated genes (SAG) or senescence enhanced genes (SEN). Their activity is generally linked to protein and/or lipid degradation. For example SAG101 codes for acyl hydrolase and the activity of SEN3 is linked to the degradation of leaf tissues (Buchanan-Wollaston et al., 2003). Proteins such as RuBisCO, which is the key protein for carbon fixation during photosynthesis, are also degraded during the deterioration of leaf cells. ROS can be responsible for such degradation. However, the activity of protease genes involved with the degradation of RuBisCO was also found to increase during cell senescence in leaves. Several other genes that encode for enzymes are responsible for the degradation of membrane lipids during cell deterioration. Whether the activity of the many genes involved with cell senescence is directly causing cell deterioration or not remains unclear. This is because some of the genes that are active during cell deterioration are likely to be directly involved in such deterioration whereas others are likely to be involved in regulatory mechanisms counteracting deterioration (Buchanan-Wollaston, 1997). Whatever the scenario, detecting the activity of such genes implies that cell deterioration is underway because the cell is becoming older.

## GENES INVOLVED WITH ORGAN SENESCENCE

Many genes that are involved in the senescence of plant cells are also found to play a role in organ senescence (**Table 1**). This is partly because many studies on organ senescence investigate how leaf tissues deteriorate with leaf age at the cellular level (e.g., ROS accumulation; Thomas and Stoddart, 1980; Smart et al., 1995; Gan and Amasino, 1997; Bleecker, 1998). Leaf senescence intervenes right after growth and involves the deterioration of the mesophyll from the tip to the base of the leaf (Jansson and Thomas, 2008). Regulatory gene activity was found to vary between the early growth of the organ and its death (Guo and Gan, 2011). MicroRNAs such as miR156 were identified in primordia of rice leaves where they target transcription factors of the SQUAMOSA PROMOTER BINDING-LIKE (*SPBL*) family and affect the duration of juvenile leaf character expression. The interplay of those MicroRNAs with the expression of miR164 was also found to affect age related cell death in *Arabidopsis* (Kim et al., 2009).

**Table 1:** Genes that are known to play a role in organ senescence (compiled from Bleecker, 1998; Thomas et al., 2009; Thomas, 2013, and references therein).

Gene	Related function	Reference
*Arabidopsis* RCCR (Red Chlorophyll Catabolite Reductase)	Chlorophyll degradation	Wüthrich et al. (2000), Pružinská et al. (2007)
*Arabidopsis* PaO	Chlorophyll degradation	Oshio and Hase (1969), Rodoni et al. (1997)
*Arabidopsis* Sgr1 (Stay green)	chlorophyll degradation	Guiamet and Giannibelli (1996), Armstead et al. (2006), Park et al. (2007), Sato et al. (2007)
*Arabidopsis* Wrky53	Cell and tissue specialization	Miao et al. (2004), Uauy et al. (2006)
*Arabidopsis* AtNAP	Cell and tissue specialization	Guo and Gan (2006)
*Auxenochlorella* dee4 – amino acid permease	Cell and tissue specialization	Hörtensteiner et al. (2000)
*Arabidopsis* Fibrillin	Cell and tissue specialization	Yang et al. (2006), Brehelin et al. (2007)
*Arabidopsis* Carotenoid Cleavage Dioxygenase8	Plastid trans-differentiation	Snowden et al. (2005)
*Brassica* dnaJ chaperone OrI	Plastid trans-differentiation	Lu et al. (2006)
*Arabidopsis* dnaJ chaperone OrII	Plastid trans-differentiation	Giuliano and Diretto (2007)
*Zea* Bronze1	Vacuole function	Furtek et al. (1988)
*Zea* myb C1 anthocyanin TF	Vacuole function	Paz-Ares et al. (1990)
*Arabidopsis* AtMRP2 ATP transporter	Vacuole function	Hinder et al. (1996), Lu et al. (1998)
*Zea* See2	Vacuole function	Smart et al. (1995), Carter et al. (2004)
Squamosa Promoter Binding-Like (SPBL) gene family	Leaf development	Poethig (2010), Bergonzi and Albani (2011), Wang et al. (2011), Xie et al. (2012)
TFL1 (Terminal Flower 1)	juvenile period in perennials	Bergonzi and Albani (2011)
SnRK1 (Snf1-Related Kinase1	Sugar mediated regulation of aging	Thelander et al. (2004), Baena-Gonzalez et al. (2007)
Hexokinase1 (HXK1)	Sugar mediated regulation of aging	Jongebloed et al. (2004), Parrott et al. (2007), Smeekens et al. (2010)
*Arabidopsis* “old” mutant	Sugar mediated regulation of aging	Schippers et al. (2008)
*Arabidopsis* AtMYB2	Bud outgrowth during monocarpic senescence	Guo and Gan (2011)
*Arabidopsis* TERT (Telomerase Reverse Transcriptase)	Telomere attrition	Watson and Riha (2010)
RTBP1 (Rice Telomere Binding Protein) 1	Telomere attrition	McKnight and Shippen (2004), Hong et al. (2007)
ATGs (Autophagy genes)	Cell degradation	Thompson et al. (2005)
TOR (Target of Rapamycin)	Growth and development	Blagosklonny and Hall (2009)

At the level of the leaf organ, the assimilation and storage of carbon and nitrogen during leaf development stops when leaf senescence starts. It is important to note that the timing of leaf senescence depends on leaf age and on the environmental resources available (Wingler et al., 2006). Leaves start their life by being a sink for the products of photosynthesis and accumulate Carbon and Nitrogen until their function changes when senescence starts. At that time, they stop being a sink and become a source of Carbon and Nitrogen for the rest of the plant. Leaves reach an advanced stage of deterioration when they transfer the products of photosynthesis to the whole plant. The shift toward leaf senescence is associated with the increased transcription of photosynthesis-associated (*PAG*) genes (Hensel et al., 1993). A whole network of regulatory functions associated with genes was found to be active during leaf senescence (e.g., CWinv, HXK1, TOR, and SnRK1, see Thomas, 2013). Other genes such as the *HYS1/CPR5* have also been identified because they are activated during leaf senescence but they are in fact counteracting the deterioration (Yoshida et al., 2002). Delayed leaf senescence was also found in *etr1-1* mutant plants. The specificity of those plants is to be that are insensitive to the plant hormone ethylene (Grbić and Bleecker, 1995). Furthermore, many other SAG and SEG genes were identified using mutant lines and Next Generation Sequencing methods (see Buchanan-Wollaston et al., 2003).

It remains unclear whether most genes involved with leaf senescence or organ senescence are directly part of a genetic program coding for senescence at these biological scales. One alternative scenario is that they exhibit an activity that is associated with the degradation of organs because they are participating to genetic mechanisms that are in fact counteracting degradation (Nam, 1997). However, whatever the reasons, the activity of these genes can therefore be used to detect organ senescence. Whether those genes are truly involved with organ senescence or are part of a developmental program of organ turnover throughout the plant life also remains unclear. It is interesting to note that leaf characteristics (defense, photosynthesis and the age at which they fall) have evolved under domestication (El-Sharkawy, 2004; Mondolot et al., 2008; Pujol et al., 2008b). These results suggest that leaf senescence may be integrated in the evolution of senescence at the whole plant level. It is nevertheless difficult to figure out how those genes can help infer how senescence affects the whole plant (Bleecker, 1998). Indeed, many of the genetic mechanisms presented above are shared by plants characterized by completely different life histories such as annual plants, short lived perennial monocarpic plants (i.e., semelparous: a single reproductive episode precedes death) and long lived perennial polycarpic (i.e., iteroparous: multiple flowering events throughout a lifetime) plants (Munné-Bosch, 2008).

## GENES PLAYING A ROLE IN WHOLE PLANT SENESCENCE

Whether the activity of the genes and the epigenetic changes presented below are relevant to the study of senescence at the whole plant level is at the center of a debate between, on one hand, physiologists, and system biologists, and in the other hand, demographers, and evolutionary biologists. How to define senescence at the level of the whole plant? Is it chronological age and/or a suite of developmental stages that lead to mortality? This is not a simple question because those two chronological factors are often confounded. Physiologists and system biologists study senescence by analysing developmental modifications. Demographers and evolutionary biologists study senescence by assessing the age trajectory of fitness components. The genetic mechanisms presented below play a role in the chronological succession of developmental stages. A doubt therefore remains on the direct relationship between the activity of those genes and age. At the level of the whole plant, *TERMINAL FLOWER 1* (*TFL1*) genes are involved with developmental changes. *TFL1* genes are suspected to play a role in senescence because they regulate the length of the juvenile period in perennial plants. The life stage-dependent activity of those genes affects, albeit only indirectly, the expression of the *LEAFY* genes and the *APETALA 1* genes that determine the development of flowers from meristematic tissue (Ratcliffe et al., 1998). In *Arabidopsis*, the *AGAMOUS* gene, which is known to play a crucial role in the development of flowers, was only found to be active during the flowering period (Yanofsky et al., 1990). Other genes are known to be responsible for an increased regulation of branching during successive developmental phases from germination to death in *Arabidopsis* (Guo and Gan, 2011). Epigenetic methylation may also play a role in the whole plant senescence. Changes in DNA methylation that drive the silencing of MuDR transposable elements were indeed observed during transitions between life stages in maize (Li et al., 2010). Furthermore, the global level of DNA methylation was also found to vary across life stages in the giant redwood tree (Monteuuis et al., 2008). It would be a great breakthrough to determine whether the chronological genetic and epigenetic changes documented above are directly playing a role in the genetic programming of senescence. To this aim, it would be necessary to disentangle age and stage specific organismal changes (Roach, 1993; Caswell and Salguero-Gómez, 2013).

## HOW TO PREDICT SENESCENCE AT THE INDIVIDUAL LEVEL

Senescence at the level of the whole organism, be it a plant or an animal, affects complex physiological, and reproductive functions. Complex characters often depend on a large genetic basis formed by more than a few genes. Quantitative genetics offers an opportunity to analyze the genetic architecture of complex traits. Using quantitative genetics to identify the signature of senescence may therefore prove useful in plants. To understand the value of using quantitative genetics in order to identify senescence at the level of the whole plant, one must go back to the basics of the evolutionary theory of senescence. In plants, as in animals, the deterioration with age of fitness related characters can evolve if older individuals contribute less in the gene pool of the next generation than younger individuals. As first suggested by Fisher (1930) and Haldane (see Medawar, 1952, p. 3), selection against age related malfunctions is not efficient if the underlying genes have already been transmitted to the next generation. This idea was further developed by Medawar (1952) and Williams (1957) who discussed the underlying genetic mechanisms. At the level of the genetic architecture of fitness related characters, theory predicts that the deleterious mutations responsible for senescence and expressed in old age will accumulate in the genome (“Mutation Accumulation” theory) as selection fails to remove them (Medawar, 1952). Another non exclusive expectation regarding the genetic architecture of senescence is that genes coding for a fitness advantage in young age, but having detrimental effects in older age (“Antagonistic Pleiotropy” theory) will be favored by selection (Williams, 1957).

In Hamilton (1966) claimed that the evolution of senescence could be universal. However, in plants, a recent analysis of demographic data bases showed that most plant species were not affected by a decay in fertility and by an increase in the risk of mortality with age (Baudisch et al., 2013). The age dependent decrease of the individual probability of survival that is expected in the case senescence was nevertheless found to be common in perennial plant species (Silvertown et al., 2001). Furthermore, longitudinal demographic surveys show that demographic senescence can occur in wild plant populations (Picó and Retana, 2008; Roach et al., 2009; Roach, 2012). Whether the demographic assumptions underlying Hamilton’s claim apply to most plants is still debated. The evolution of senescence does not depend only on survival. Reproductive success is also a key parameter that must be taken into account because it molds the relative contribution of individuals to the gene pool of the next generation (Partridge and Barton, 1993). In a scenario where a higher reproductive success is favored in younger age, theory also predicts that senescence will evolve.

In the 1990s, Roach inventoried cases of indirect phenotypic evidence suggesting that senescence had evolved in *Papaver*, *Solidago*, *Poa*, and *Geranium* plant species (Roach, 1993). At the genetic level, two theories (Mutation Accumulation and Antagonistic Pleiotropy) predict specific modifications of the additive genetic variability of fitness traits with age (Flatt, 2012; Rose et al., 2012). These predicted changes are the consequence of the failure of natural selection to remove genes with deleterious effects in old age (Greer, 2012). This is because such genes were already transmitted to the next generation. Under the evolutionary theory of senescence, age is correlated with a decline in fitness. We also expect aging rates to be genetically based. Finally, both the mutation accumulation and antagonistic pleiotropy mechanisms are expected to drive an increase in additive genetic variance with age. Negative genetic correlations between early and late age classes are expected to bring evidence for antagonistic pleiotropy. In animals, especially in birds, and ungulates, quantitative genetics was used to confirm these expectations already several times (Charmantier et al., 2006a,b; Brommer et al., 2007; Kruuk et al., 2008; Monaghan et al., 2008; Nussey et al., 2008; Wilson et al., 2008). It is likely that age dependent specific patterns of quantitative genetic variation, in other words Gene by Age interactions (G × A), can also be used to detect senescence in plants.

## THE EVOLUTIONARY QUANTITATIVE GENETICS OF SENESCENCE IN PLANTS

Pujol et al. (2014) detected the quantitative genetic signature of senescence in *Silene latifolia*, a short lived perennial plant species. They found that the reproductive performance of individuals was declining with age. They also found that the age dependent deterioration of reproduction had an additive genetic basis. Finally, they found that additive genetic variation for reproductive characters increased with age, which is predicted under both Mutation Accumulation and Antagonistic Pleiotropy hypotheses. However, they did not find negative genetic correlations between young and old age classes as expected under the Antagonistic Pleiotropy hypothesis. This investigation was rendered possible by the use of a pedigree based random regression animal model which is a method first designed to estimate the quantitative genetic variation of phenotypic plasticity (Wilson et al., 2005; Nussey et al., 2007b; Brommer et al., 2008; Pujol and Galaud, 2013). Such finding calls for the replication of such studies in other plant species across the plant kingdom.

Roach (1993) was already calling for investigating the quantitative genetic architecture of senescence in plants. Shefferson and Roach (2012) found evidence for genetic variation in population growth rates. There is now evidence that variation in individual aging rates has a genetic basis (Pujol et al., 2014). Why are such studies so rare? First, the longitudinal surveys that are necessary for such studies are very demanding. Those surveys are labor and time intensive and require large sample sizes. Another non exclusive explanation is that research projects on plant senescence are often investigating how plants can escape senescence rather than analyzing the senescence process in plants that are negatively affected by aging. Already Harper (1977) was mentioning that plants did not show apparent senescence as a consequence of their indeterminate growth. Ever since then, many efforts were logically directed toward identifying what could cause plants to avoid senescence (Watkinson, 1992; Orive, 1995; Pedersen, 1995; Gardner and Mangel, 1997; Vaupel et al., 2004; Munné-Bosch, 2008; Garcia et al., 2011; Martínez, 2012; Morales et al., 2013; Salguero-Gómez et al., 2013). As a consequence, it is not surprising that the quantitative genetic signature of senescence may have been overlooked in plants. Some insights could certainly be gained by comparing the effect of age on the quantitative genetic basis of fitness related traits in species whose demography and/or physiology suggest that senescence is absent.

Knowledge on additive genetic variation at fitness related traits can be used to predict the evolutionary potential of a population (Etterson, 2004; Pujol and Pannell, 2008; Pujol et al., 2008a; Dibattista et al., 2009; Pujol et al., 2010). This is because additive genetic effects are expected to underlie most phenotypic evolutionary changes under selection (Hill et al., 2008). Today, methods are available to describe the age dependent additive genetic variation of fitness related traits in animals and plants (Charmantier et al., 2006a,b; Brommer et al., 2007, 2010; Nussey et al., 2007a, 2008; Wilson et al., 2007; Monaghan et al., 2008; Wilson, 2008; Morrissey et al., 2010, 2012; Pujol et al., 2014). Knowing such age dependent patterns has implications for the study of the adaptive potential of populations because populations that are characterized by different age structures have different evolutionary properties (Ronce and Promislow, 2010).

It is important to note that genes with additive effects on the phenotype are probably not the only genes forming the genetic architecture of senescence. Ignoring other components of the genetic architecture of fitness related traits such as dominance and epistasis may result in missing an important part of an evolutionary process (Barton and Turelli, 1989; Fenster et al., 1997; Barton and Keightley, 2002; Wilson and Reale, 2006; Reid et al., 2011; Hansen, 2013; Mackay, 2013). Quantitative genetics models including dominance effects have already been proposed (e.g., Wolak, 2012; Vitezica et al., 2013; Sun et al., 2014). An alternative way to approach dominance is to study inbreeding depression because it is the expression of recessive deleterious mutations. Under the Mutation Accumulation hypothesis (Medawar, 1952), inbreeding depression should depend on age (Charlesworth and Hughes, 1996). In a landmark meta-analysis of the magnitude and timing of inbreeding expression in plants, Husband and Schemske (1996) conjectured that the accumulation of late-acting inbreeding depression usually found across plant taxa was likely the result of the expression of mildly deleterious recessive mutations that could not be purged by natural selection (because the mutations had small effects). However, the patterns they observed are also consistent with a weaker action of selection with age as expected under the evolutionary theory of senescence. The magnitude of inbreeding depression was often compared among populations and environments (Husband and Schemske, 1996; Pujol and McKey, 2006; Pujol et al., 2009; Facon et al., 2011). It would be insightful for the understanding of the evolution of senescence in plants to accumulate case studies that compare inbreeding depression between age classes in the litterature.

Environmental variability can interact with genetic variability to shape the phenotype (Wilson et al., 2005; Kruuk and Hadfield, 2007; Nussey et al., 2007b; Pujol and Galaud, 2013). However, only few studies quantified how the environment interacts with the genetic architecture of senescence (Nussey et al., 2007a). In their study, Nussey et al. (2007a) found that age did interact with both quantitative genetic variation and environmental variation. Their findings imply that environmental constraints have the potential to amplify the magnitude of senescence. It would be worth testing whether such results can be found in other taxa and correspond to a general property of senescence. Also, age dependent epigenetic changes were found to be associated with senescence (Li et al., 2010). It is now widely acknowledged that epigenetic variation can be transmitted to the next generation and that such inheritance system can influence phenotypic evolution (Danchin et al., 2011; Mesoudi et al., 2013; Singh et al., 2014). Since quantitative genetic statistical models can also accommodate such non genetic source of transgenerational variation (Day and Bonduriansky, 2011; Danchin et al., 2013; Townley and Ezard, 2013), it would be worth verifying whether senescence related expectations on genetic variation also apply to transgenerational epigenetic variation.

## TOWARD AN INCLUSIVE GENETIC THEORY OF SENESCENCE

**FIGURE 1:**
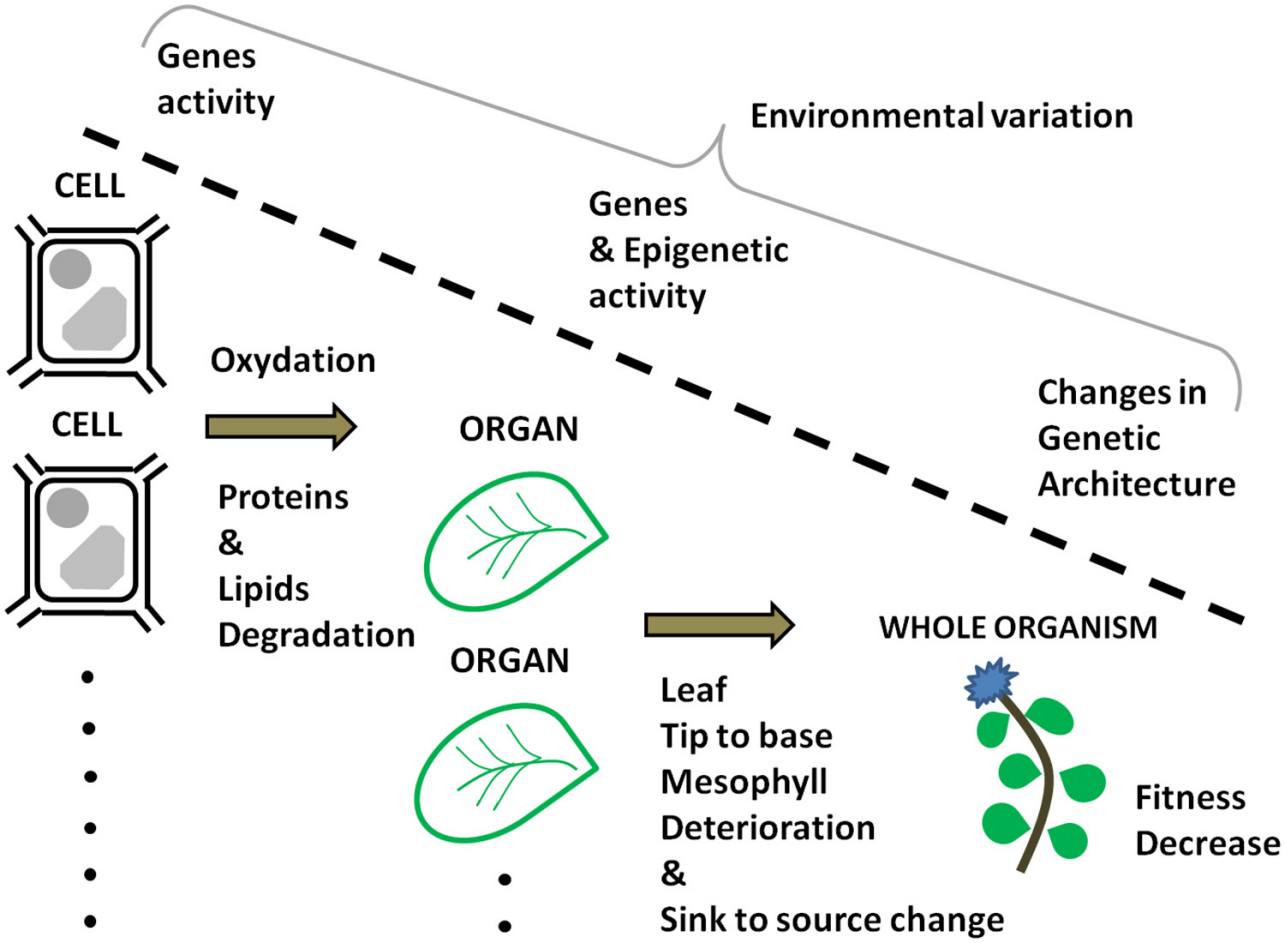
**Age-dependent information at different levels of organization that can be integrated within a quantitative genetics framework as to identify their relationship**.

At present, prediction of plant senescence based on geneti and environmental data is hampered by the lack of integration between the scales at which senescence is studied, i.e., cells, organs, and individuals (**Figure 1**). One if not the most important challenge for the future is to understand the genetic interactions between those scales. Are the genes involved with senescence at the cell and the organ level similar to those that are affected by the increase with age of deleterious mutations balanced by selection? Quantitative genetics offers an opportunity to advance in that direction. Quantitative genetics provides us with the framework to predict the phenotype of individuals on the basis of our knowledge of their genetic architecture (Falconer and Mackay, 1996; Roff, 1997; Lynch and Walsh, 1998; Mackay, 2001). The usual approach in quantitative genetics is to use statistical models in order to relate phenotypic resemblance to genetic similarities. Those genetic similarities can be established on the basis of pedigree information and/or molecular genomic data (Abney, 2008; Carré et al., 2013; Gay et al., 2013; Bérénos et al., 2014). This framework allows us to accommodate multi-trait analyses, longitudinal analyses, and nested genetic effects (Lande and Arnold, 1983; Roff, 1996; Delph et al., 2004, 2005; Etterson, 2004; Clements et al., 2011; Teplitsky et al., 2014). As a result, age-dependent variation in cell, organ, and individual characteristics can be adequately studied using models accommodating longitudinal data such as a random regression animal models (Fischer et al., 2004; Schaeffer, 2004; Wilson et al., 2005; Nussey et al., 2007b; Brommer et al., 2008; Pujol and Galaud, 2013). Such models can evaluate genetic effects at different biological scales by including multi-trait and nested variables. In this way, it is possible to study the dynamics of senescence and the genetic correlations among the genetic components of senescence at multiple biological scales. Only then will we be able to test the hypothesis that cellular, organ, and individual senescence are interdependent (Bleecker, 1998) and to bring an answer to the question: are genes underlying senescence at multiple biological scales belonging to the same genetic program that was shaped by the failure of natural selection in old age? Access to genome wide decrypted information may help answering that question. The combination of next generation sequencing techniques to novel methodological developments in quantitative genetics (Beraldi et al., 2007; Brown et al., 2012; Gay et al., 2013; Bérénos et al., 2014) has a lot of unexploited potential for the genetic study of senescence. Using those methods, DNA sequence variation data can be used to quantify how molecular polymorphism is expressed in different compartments (cells, organs, etc.) in function of the age of the plant. Collectively, the elements presented above illustrate how the genetic knowledge on senescence is scattered across separated research domains. The time is ripe to integrate those disciplines in one approach that will include multiple biological scales. Only then will we be able to identify the relationship between the genes involved with senescence at the cell, organ, and individual levels (**Figure 1**).

## CONCLUSION

The genetic prediction of senescence in plants can be seen as an equation. The genetic architecture of a plant would be on one side of that equation. On the other side of this multivariate equation would be the age dependent deterioration of cells, organs, and functions that define the fitness performance of plants. The reviewed elements presented above imply that several links between the two sides of the equation do exist. However, this equation remains incomplete. As I underlined above in various sections of the text, many genetic, and environmental effects remain to be investigated. It is also crucial to identify the genetic links between the biological scales at which organismal functions decline. Quantitative genetics offers a unique opportunity to build such an integrative framework. Understanding the genetic architecture of senescence in plant populations is equivalent to evaluating the adaptive potential of fitness changes with age. Such studies should therefore be an asset for the management of plant genetic resources and the conservation of endangered plant species.

In fact, attempts to reconcile the separated components of the genetic architecture of senescence in plants are scarce. The potential implications of such studies conducted across the plant kingdom in the light of the immense variation it conceals in terms of genomic complexity, life spans, reproductive systems, and life histories are immense. In plants, it is easier to find closely related species that display large phenotypic resemblances but have very different life histories. Genetic studies on senescence in animals and humans are a top research priority that has a direct interest for the people (Zimniak, 2012; Yashin et al., 2013). It is understandable that the interest for these studies outweighs the interest for genetic research on plant senescence. One should nevertheless not neglect that adopting an inclusive approach of senescence in plants that integrates multiple biological scales would bring alternative insights on issues raised by aging research in animals and humans.

## Conflict of Interest Statement

The author declares that the research was conducted in the absence of any commercial or financial relationships that could be construed as a potential conflict of interest.
